# Complete reannotation of the *Arabidopsis *genome: methods, tools, protocols and the final release

**DOI:** 10.1186/1741-7007-3-7

**Published:** 2005-03-22

**Authors:** Brian J Haas, Jennifer R Wortman, Catherine M Ronning, Linda I Hannick, Roger K Smith, Rama Maiti, Agnes P Chan, Chunhui Yu, Maryam Farzad, Dongying Wu, Owen White, Christopher D Town

**Affiliations:** 1 The Institute for Genomic Research, 9172 Medical Center Drive, Rockville, Maryland, 20850, USA

## Abstract

**Background:**

Since the initial publication of its complete genome sequence, *Arabidopsis thaliana *has become more important than ever as a model for plant research. However, the initial genome annotation was submitted by multiple centers using inconsistent methods, making the data difficult to use for many applications.

**Results:**

Over the course of three years, TIGR has completed its effort to standardize the structural and functional annotation of the *Arabidopsis *genome. Using both manual and automated methods, *Arabidopsis *gene structures were refined and gene products were renamed and assigned to Gene Ontology categories. We present an overview of the methods employed, tools developed, and protocols followed, summarizing the contents of each data release with special emphasis on our final annotation release (version 5).

**Conclusion:**

Over the entire period, several thousand new genes and pseudogenes were added to the annotation. Approximately one third of the originally annotated gene models were significantly refined yielding improved gene structure annotations, and every protein-coding gene was manually inspected and classified using Gene Ontology terms.

## Background

*Arabidopsis thaliana *has long been considered the foremost model organism in plant biology. It is favored for its short generation time, plentiful seeds, conveniently small stature, and ease of genetic transformation using *Agrobacterium tumefaciens*. Its comparatively small genome size, estimated at 140 million base pairs, and low repetitive sequence content drove the choice of *Arabidopsis *as a target for complete genome sequencing in the early nineties. Ten years later, the genome sequence was completed [1], providing a valuable resource for furthering the understanding of *Arabidopsis *biology and providing a reference sequence from which results in *Arabidopsis *could be extended to other plants.

Since its publication, the *Arabidopsis *genome has been mined for clues to numerous important metabolic pathways and biological processes, many of which are documented in peer-reviewed publications including the *Arabidopsis *Book [2]. Additionally, the *Arabidopsis *genome has been used extensively as a tool for comparative genomics, both for genome-wide comparisons and to study specific processes among a wide range of plant species, including the gametophytic transcriptome of mosses [3], wood and secondary cell wall formation in woody gymnosperms [4], and legume symbiosis [5].

Unlike the genomic sequence, which is mostly unambiguous and unlikely to change significantly over time, the genome annotation is dynamic and expected to improve further as we better understand the molecular biology of *Arabidopsis *and related plants. The original *Arabidopsis *genome annotation that accompanied the completed genome sequence in 2000 [1] represents the earliest comprehensive depiction of gene content and predicted gene functions. This original annotation was accumulated over the course of the sequencing effort in the form of individually annotated BAC sequences submitted to GenBank by each of the sequencing centers. Due to the diversity of annotation tools and protocols employed by participating centers during this process, and continuing improvements in annotation resources over the several years of the sequencing project, preliminary gene annotations varied considerably in accuracy and quality at the level of both gene structure and gene function. This heterogeneity within the annotation was most visible in the context of gene families constructed upon completion of the entire genome sequence. Related genes often had dissimilar names and predicted functions as well as incongruent gene structures. A coordinated effort was needed to provide a more useful resource to the plant scientific community.

Immediately after the initial data release, The Institute for Genomic Research (TIGR) began a reannotation effort [6], with the goal of improving the annotation by refining gene structure and gene function assignments, employing the latest annotation tools and resources, and applying uniform annotation protocols across the entire genome. Over the course of this reannotation effort, which lasted three years and ended in January 2004, five milestone annotation releases were generated and provided to the public by TIGR, hosted additionally by the National Center for Biotechnology Information (NCBI) and The *Arabidopsis *Information Resource (TAIR). The fifth annotation release (January, 2004) represents our final major contribution to the *Arabidopsis *genome reannotation effort and is the main focus of this manuscript.

The primary goals of this reannotation are summarized as follows:

• refine gene structures, including the annotation of alternative splicing variants and untranslated regions (UTRs);

• manually review gene names and assign genes to Gene Ontology [7] controlled vocabularies describing molecular function, biological process and cellular location;

• recreate chromosome sequences accurately, depicting the genome based on the most current BAC tiling path.

Here we present a summary of our annotation methods, efforts and history leading to the fifth and final TIGR release of the *Arabidopsis *genome annotation.

## Results and discussion

### Contents of *Arabidopsis *genome annotation release 5

The final TIGR genome reannotation release contains annotations for 26,207 protein-coding genes, 631 tRNAs, 2 rDNA cassettes (18S, 5.8S and 25S rDNA units), 57 snoRNAs, and 15 snRNAs (Table 1). Of the 26,207 protein coding genes, 2,330 are annotated with alternative splicing isoforms and 18,099 are annotated with UTRs. Genomic regions with homology to open reading frames (ORFs) of transposable elements (2,355) and pseudogenes (1,652) account for an additional 3,786 annotations, and (in contrast to earlier releases) are now separated from the total protein coding gene count.

Taking into account alternative splicing variants, the 26,207 protein-coding genes yield 27,855 distinct protein sequences. Nearly 85% of these proteins contain a match to an InterPro [8] accession via PROSITE [9], ProDom [10], PRINTS [11], Pfam [12] or TIGRFAM [13], and nearly 30% are predicted by TMHMM [14] to contain at least one transmembrane domain.

**Figure 1:**
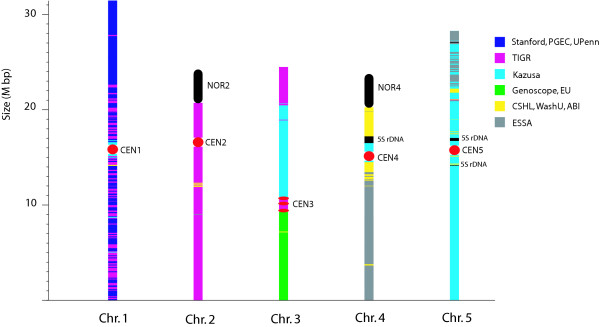
The *Arabidopsis *genome as depicted in release 5 of the *Arabidopsis *genome annotation. Each BAC sequence region within each chromosome is shown colored according to the original sequencing group. The unsequenced NOR and 5SrDNA clusters are colored black and centromeric regions are colored red, both with rounded edges and drawn to scale based on their estimated sizes.

The *Arabidopsis *genome sequence is essentially complete. The representation of the *Arabidopsis *genome sequence as provided in release 5 is illustrated in Fig. 1. The sequenced portion of the *Arabidopsis *genome now stands at approximately 119 Mbp, including sequences from 1,611 tiled BACs, PACs, YACs, cosmids and PCR products. Unsequenced regions of the genome are restricted to the centromeres of each chromosome, 5S rDNA clusters on chromosomes 4 and 5, and the nucleolar organizer regions (NOR) at the northern ends of chromosomes 2 and 4. With the exception of the NORs and the northern tip of chromosome 5, every other chromosome terminates with either perfect copies of the telomeric repeat (AAACCCT), or degenerate copies of this sequence that are characteristic of sub-telomeric regions. These repeats are found inverted at the bottom of chromosome 3. The regions of overlap between adjacent BACs in each chromosome tiling path were reviewed extensively during our reannotation effort, and the chromosome sequences were generated based on the joining of regions of BAC sequences to yield our most accurate depiction of contiguous chromosomes. A series of 1000 'N' characters were inserted into the chromosome sequence at positions representing the unsequenced regions described above, only to provide placeholders for the unsequenced components. The centromere of chromosome 3 includes two internal sequenced contigs each flanked by unsequenced regions. In addition, partially sequenced BACs mapped to centromeric locations are included in both the chromosomal tiling paths and in the representation of the chromosome sequence in order to provide the most comprehensive sequence data possible.

How complete a representation of the genome is the version 5 tiling path and pseudomolecules? In the sequencing phase of the *Arabidopsis *Genome project, it was agreed that each group would continue sequencing up to the region containing intractable centromeric repeats. In order to make the public version of the genome as complete as possible, centromeric BACs for which sequencing was still in progress but the position of which in the tiling path was known were included in builds of pseudomolecules. These sequences are not included in the genome annotation and consist mainly of transposon-related and other centromere-associated sequences. A minimal estimate of the extent of the genome within the centromeres is ~1 Mb per centromere [15] although a recent new estimate of genome size [16] could indicate that the amount of unsequenced genome is larger than this. As reported previously [6], survey sequencing of representative centromeric BACs revealed no firm evidence for previously undetected genes in the centromeric regions.

A second view of genome completeness comes from an assessment of the representation of *Arabidopsis *ESTs in the genome sequence. After removal of contaminating human and *E. coli *sequences, approximately 2% of all ESTs did had no cognate match in the genome sequence [6]. Investigation of 20 of these "missing genes" by PCR on genomic DNA revealed that only 3 could be detected and all were organellar in origin.

## Improvements in the annotation from release 1 through 5

Each annotation release represents one or more milestones within our reannotation effort, providing key contributions towards annotation improvement. These are summarized below and elaborated upon in subsequent sections:

• Release 1 (August 2001).

• The incorporation and assimilation of non-TIGR BAC sequences and annotations into the TIGR ATH1 Sybase relational database.

• TIGR XML format was developed and applied to represent the structured contents of ATH1 for public use.

• Release 2 (January 2002)

• Approximately 5,000 full length (FL) cDNAs from Ceres, Inc. were incorporated into gene models [17]

• The annotation was used as the basis for ATH1-Affymetrix *Arabidopsis *whole genome microarray chip design [18].

• Release 3 (August 2002)

• Incorporation of the RIKEN *Arabidopsis *FL-cDNA sequence collection [19] into gene structure annotations using the same methods as employed in the incorporation of the Ceres FL-cDNAs.

• Comprehensive analysis of intergenic regions using the latest gene finders, incorporating previously missed gene annotations and new hypothetical genes.

• Release 4 (April 2003)

• The development and application of the FL-cDNA and EST alignment assembly pipeline PASA, incorporating ESTs and FL-cDNAs into gene structure annotations, modeling alternative splicing variants, and maximizing UTR annotations [20].

• Release 5 (January, 2004)

• Improved annotation of transposon-homologous regions and pseudogenes.

• cDNA sequences, provided pre-publication by Genoscope [21], allowed for the annotation of an additional ~1000 alternatively spliced genes, nearly doubling the count from the previous release.

• Completion of GO assignments to all annotated genes.

The overall gene density and gene structure statistics differ little from the initial genome annotation. The statistics alone, however, fail to emphasize the improvements that have been made to individual gene annotations over the course of our reannotation effort. Direct comparisons of individual genes between each of the annotation releases provide a more accurate measure of the level of change. Updates performed on gene structures between successive releases of the annotation include modifying individual exon boundaries, splitting single gene structures into two or more genes, merging multiple gene annotations into single genes, deleting poorly supported genes, adding UTR annotations to existing gene models, and creating new gene models. In addition to structural changes, gene names were systematically refined and Gene Ontology assignments were applied. A summary of the contents and changes made between releases is provided in Table 2.

By comparing release 5 to release 1, we find that only 17,975 (67%) of the original gene structures (excluding UTR updates) remain exactly the same. There were 4,241 new genes modeled, 1,130 gene models deleted, 329 genes merged, 253 genes split, and 7,094 updates to existing gene structures. Any protein-coding genes that are still not annotated are likely to be short, to lack homology to known genes, and/or to be compositionally atypical of the majority of *Arabidopsis *protein-coding genes.

The changes in the sequenced genome size between annotation releases from 115.4 M bp to 119.0 M bp can be attributed to our refinement of the specification of BAC overlaps, the addition of newly sequenced BACs previously absent from the tiling path, the inclusion of partially sequenced centromeric BACs within the tiling path, and the replacement of partially sequenced BACs with more fully sequenced/assembled versions in subsequent GenBank releases.

### Improving gene structures

Gene structure reannotation focused on improving the accuracy of the existing gene structure components, including the refinement of exon boundaries, annotation of UTRs, and identification of alternative splicing variations and pseudogenes. This effort relied primarily on sequence homology, exploiting spliced transcript and protein alignments to infer gene structures. Improved de-novo gene predictors also proved useful in the process of reviewing the annotated gene structures, especially in regard to hypothetical genes, which lack protein homology or EST support.

#### Incorporation of full-length cDNAs and ESTs into gene structures

Our initial effort to automate gene structure improvements employed ~5,000 FL-cDNAs generated by Ceres, Inc [17]. We developed software tools for modeling genes automatically using alignments of FL-cDNAs, and performed updates to existing gene structure annotations or modeled new genes where none previously existed. FL-cDNA alignments supported structural modifications for approximately 30% of the previously annotated genes, as well as providing UTR annotations for many genes.

Our most recent effort to automate gene structure annotation improvements utilized both FL-cDNAs and EST sequences. We developed the Program to Assemble Spliced Alignments (PASA) annotation pipeline to maximally assemble alignments of FL-cDNA and EST sequences and to automatically incorporate the alignment assemblies into the existing gene structure annotations. This included updating exon structures, adding UTRs, modeling new genes, and annotating alternative splice variants where supported by the transcript alignment data [20].

Through the use of the PASA pipeline, the majority of EST and FL-cDNA alignments were incorporated into the *Arabidopsis *gene annotations. As of 10/08/2003, GenBank included 31,654 FL-cDNAs and 192,671 non-FL sequences. This data set, supplemented with a transcript sequence database from Genoscope comprising an additional 21,508 FL-cDNAs and 8,039 non-FL sequences, totaled 53,162 FL-cDNAs and 200,710 non-FL sequences. Of the 16,250 genes matching a FL-cDNA, 14,555 gene models are now consistent with the FL-cDNA alignments, integrating 43,445 of the FL-cDNAs into the gene structure annotations. In addition, 90% of the ESTs that provide high quality alignments to the genome are also incorporated into gene structure annotations. The FL-cDNAs that were not fully integrated into gene structure annotations include aberrantly spliced transcripts, antisense mRNAs, polycistronic mRNAs, mRNAs encoding short, partial or unidentifiable ORFs, mRNAs with non-consensus splice sites, and mRNAs that did not align well to the genome using the spliced alignment utilities employed. Several of these topics are elaborated upon in subsequent sections. The annotated gene structures integrating FL-cDNA sequence alignments are identified by tags ("<CDNA_SUPPORT>") in the TIGR-XML distribution of our annotation, available on our ftp site [22].

Of the 19,117 *Arabidopsis *genes matching alignment assemblies, only 2,867 (15%) lack a FL-cDNA match. Thus nearly all *Arabidopsis *genes with expression detectable using current cDNA cloning methods are currently represented by a FL-cDNA sequence. Additional sequence-based methods for ascertaining gene expression, including massively parallel signature sequencing (MPSS) and serial analysis of gene expression (SAGE), have provided evidence for approximately 450 additional expressed genes that were previously annotated as hypothetical proteins due to lack of sequence evidence of expression [23,24].

#### Alternative splicing

Alternative splicing of mRNAs has many roles that impact biological systems. Variations in protein sequence resulting from alternative splicing can result in altered structures, functions, or subcellular localizations of gene products [25-29]. Alternative splicing has been given a great deal of attention in the study of mammalian genomes and is thought to be a major factor contributing to the diversity of gene products and gene functions [30,31]. Given its potential biological significance [32], accurate annotation of alternative splicing in *Arabidopsis *is clearly important.

**Figure 2:**
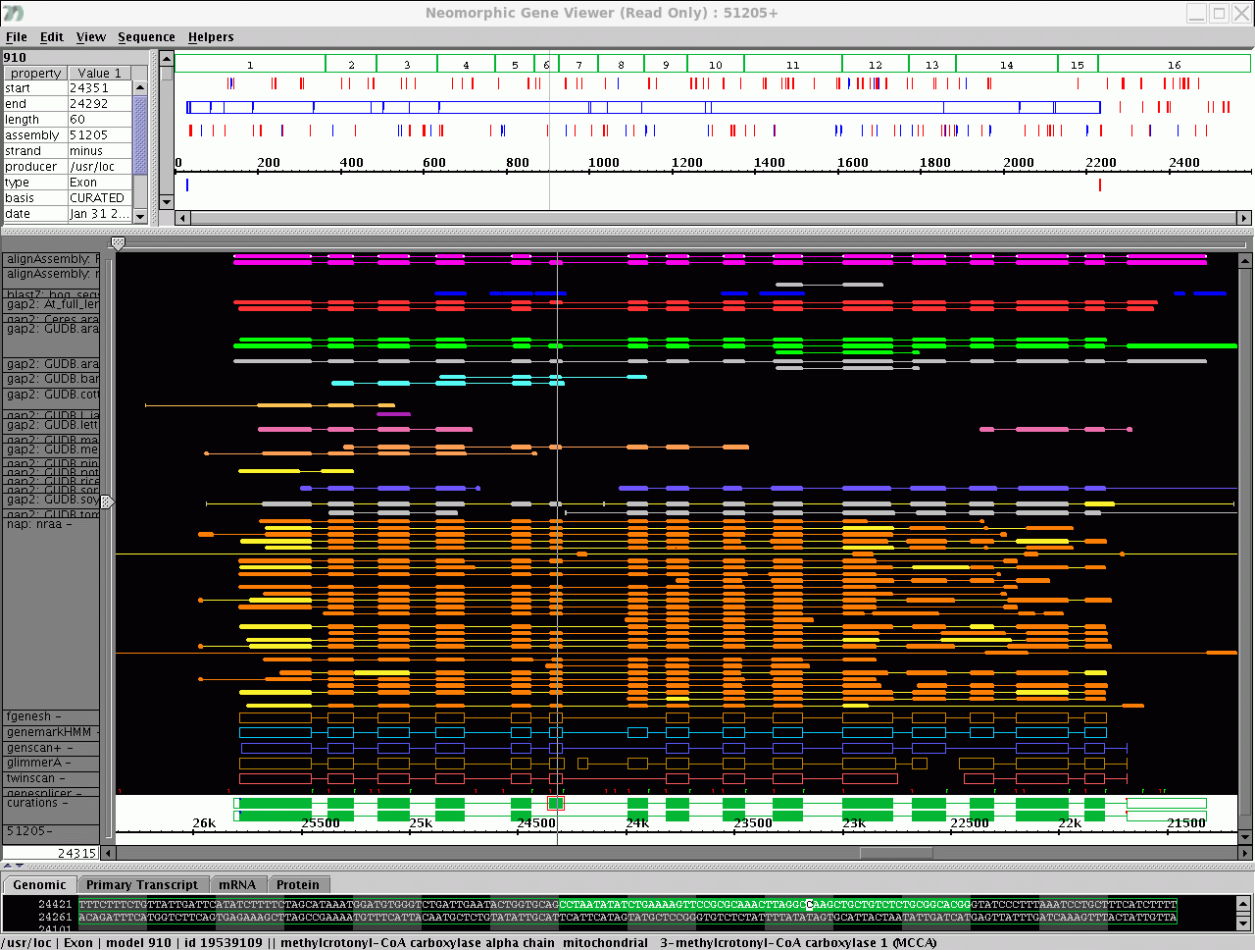
Screenshot of the Annotation Station gene editor. The evidence for gene identification and gene modeling is viewed using proprietary software called Annotation Station, developed by Neomorphic and maintained now by Affymetrix. This tool, similar to Apollo that was developed at Berkley and Sanger [105], is used by human annotators as a genome navigation tool and gene structure modeling tool. The gene models, proteins and transcript alignments are shown for an approximately 4.5 kb window along the minus strand of BAC F10O3 in the region encoding the 3-methylcrotonyl-CoA carboxylase 1 (At1g03090). The curated gene structures are shown in dark green on the white background towards the bottom of the view, with exons filled, and introns and UTRs unfilled. Above this curation within the black background, evidence is shown from bottom to top as follows: splice site predictions, computational gene predictions, protein alignments shown in orange, EST alignments from searching the various plant Gene Indices in varied colors, regions of homology to the genome of *Brassica oleracea *shown in dark blue at the top of the view, and PASA *Arabidopsis *transcript alignment assemblies at the top shown in bright pink. The vertical marker line indicates the position of a skipped exon (supported by both PASA FL-cDNA and protein alignments) that results in two protein isoforms.

Experimental investigation of splicing variations in *Arabidopsis *has been limited to a small number of genes (examples in [33-35]). Over the course of our reannotation effort, analyses of ESTs and cDNAs indicated that alternative splicing in plants is more prevalent than previously thought [17,20,36,37]. Through automated and manual methods, we have identified and annotated large numbers of splicing variations in *Arabidopsis*. Of the 26,207 protein-coding genes, 2,330 were found to have alternatively spliced forms. Comparisons between sibling transcript isoforms indicate that at least 30% of the variations result in an altered ORF yielding a non-identical protein sequence (Table 3). The remainder appear to lie exclusively within the UTR, not affecting the annotated protein sequence. Most of the alternative splicing variations are categorized as alternative donor/acceptor splice sites or unspliced introns. Relatively few examples of splicing variations involved exon skipping (and example of which is shown in Figure 2) or alternate terminal exons. Most variations affecting alternate terminal exons were restricted to the UTR regions, indicative of alternate transcriptional start and/or stop sites and presumed impacts on splicing patterns. Variations involving skipped exons tended to impact translations in a similar manner to unspliced introns and alternate acceptors/donors, although they occur much less frequently, with only 130 examples currently identified. These splicing variations would be excellent targets for further functional analyses.

#### Unspliced, antisense and dicistronic transcripts

There are numerous transcript sequences in GenBank that, when analyzed manually in the context of the genome annotation, do not appear to encode complete proteins. Many of these transcripts contain unspliced introns or indicate alternate splice sites that strongly and adversely impact the presumed correct ORF. It is not clear whether these perceptibly corrupted versions of the genes represent biologically meaningful isoforms, mistakes by the splicing machinery that are of no consequence, or artifacts of the cloning and sequencing methods employed. cDNAs with unspliced introns are often presumed to have originated from incompletely processed mRNAs. In the context of genome annotation, unspliced introns often yield stop codons and/or change the reading frame, resulting in a truncated ORF. However, many of these could be the result of regulated mRNA splicing. For example, an alternatively spliced transcript of RPS4 lacks splicing of an intron, which results in the loss of a terminal protein domain. It has been shown that this incompletely spliced isoform is biologically significant and is required, in addition to the completely spliced isoforms, for wild-type disease resistance [38]. There are 3,025 FL-cDNAs derived from 1,565 *Arabidopsis *genes that appear to result from splicing aberrations, as they are incompatible with and appear to corrupt our current representations of the full-length protein coding genes, which are presumed more accurate [39].

During the course of re-annotation, we identified 221 genes for which there are expressed sequences that align with the opposite strand and the transcribed orientation of which is confirmed by splice sites [40]; approximately half of these antisense transcripts derive from FL-cDNAs. Independent confirmation for the existence of naturally occurring antisense transcripts comes from two sources. Using an Affymetrix whole genome tiling array, Yamada et al. [41] reported the detection of antisense transcripts from ~7,600 genes. Using MPSS, Meyers et al. [23] reported the expression of antisense transcripts from 4,698 genes (4,298 exonic and 400 intronic). Although the significance of this large number of antisense transcripts in *Arabidopsis *remains to be determined, there is a growing recognition of the existence and functional significance of antisense transcripts in a variety of systems [42-44]. The order of magnitude difference between antisense transcripts recognized in cDNA/EST libraries and those detected by expression analysis and MPSS suggests that many of these transcripts are expressed at low levels and are not found in cDNA/EST libraries, or they represent unspliced transcripts that were not examined here because of a lack of confidence in the direction of their transcription.

There are at least 20 examples of mRNAs that provide transcripts corresponding to two adjacent genes [45]. Stop codons intervene and separate the two open reading frames within the transcript and, upon manual examination, it is clear that two distinct genes are represented by the single polycistronic transcript. In several cases, FL-cDNAs corresponding to the individual genes exist as well as the unexpected transcript encoding both genes. Dicistronic transcripts have previously been reported in a number of other eukaryotes including *D. melanogaster *[46,47], *C. elegans *[48-50] and *H. sapiens *[51], and in some cases have been shown to have functional significance [52]. The small number of these polycistronic transcripts identified in *Arabidopsis *is an indicator of their low frequency of occurrence. The finding of FL-cDNAs corresponding to individual genes of the polycistronic transcripts suggests that the latter may be an aberration resulting from improper transcriptional termination and polyadenylation, although a functional role has not been ruled out. In some cases, the two genes could plausibly be part of the same pathway or process for which coordinated regulation might be advantageous. Examples of genes found here as dicistronic transcripts include H+-transporting two-sector ATPase (At2g25610) and protein phosphatase 2C (At2g25620), prenylated rab acceptor (PRA1) family protein (At3g13710) and putative RNA-binding protein (At3g13700), and putative UDP-glucose 4-epimerase (At4g10960) and lipase class 3 family protein (At4g10955). Studies are needed to ascertain their significance.

#### Other plant ESTs and homologous protein alignments

The high-quality, near-perfect transcript alignments of *Arabidopsis *cDNAs/ESTs to their cognate genomic sequence proved largely amenable to automated incorporation into the genome annotation. Lower quality alignments of homologous FL-cDNA and EST sequences from other plants as well as spliced alignments of homologous proteins also served as excellent sources of data from which to infer gene structures. However, they were not as easy to incorporate computationally given that the reliability of the alignment data often varies considerably across their extent. Thus, identification of gene structures conflicting with these spliced alignments was performed automatically, but updates to individual genes based on these spliced alignments were carried out manually using Neomorphic's Annotation Station (Figure 2) (Neomorphic was acquired by Affymetrix on 10/31/2000). Genes supported only by homologous proteins or cDNAs/ESTs derived from other plants can be retrieved at [53].

#### Comprehensive gene discovery employing gene prediction tools

Gene prediction programs have been useful in identifying potentially novel genes, as well as missed or incorrect exons. In the original *Arabidopsis *genome annotation, several genomic regions lacked comprehensive gene identification possibly due to the shortcomings of the programs employed. The operational criterion for instantiating a gene model in the *Arabidopsis *genome is for a gene structure to be predicted similarly by two different gene-prediction programs. With our latest set of gene prediction programs including GENSCAN+ [54], GeneMark.hmm [55], and glimmerA (glimmerM variant trained for *Arabidopsis *[56]), we applied this criterion to all genomic regions annotated as intergenic, automatically creating new genes within each region as the minimal criterion was satisfied. To avoid the spurious promotion of numerous small gene predictions, many of which are likely to be false positives, a conservative minimum protein length cutoff of 110 residues was applied in this automated process. This was chosen conservatively to reflect the 5th percentile of the protein length distribution derived from the previously existing, manually curated *Arabidopsis *protein-coding gene annotations.

Since previous releases of the annotation lacked the comprehensive annotation of transposon-homologous regions, many intergenic regions were found to harbor gene predictions that matched transposon ORFs. These gene models were specifically excluded from the final round of automated gene modeling and were addressed separately. Through our analysis of intergenic regions we annotated 785 new genes, of which 665 had homology to other proteins. The remaining 120 genes were annotated as additional hypothetical genes. The newly annotated genes with homology to known sequences indicate the significant number of gene annotations missed in the original genome annotation. Thus, improved gene prediction programs and increased database content provided us with an additional set of genes worthy of incorporation into the genome annotation and further study.

#### Manual refinement of gene structures

Throughout the reannotation project, significant effort has been focused on manually refining intron and exon boundaries of gene models predicted by the various automated processes. Initially, the team of 4–6 annotators would progress along BAC sequences and correct, add and delete gene models as necessary. Later, the annotators assessed pre-computed gene families for consistent gene structures concurrent with functional annotation (described below).

Intron-exon boundary refinements and UTR additions were performed by annotators viewing alignments generated by the Eukaryotic Genome Control (EGC) computational pipeline (see methods) using the Annotation Station graphical user interface (Figure 2).

### Gene function annotation

The primary goal of the functional annotation effort was to produce a high quality, consistently named proteome. The results from numerous bioinformatics analyses such as homology matches and domain hits were made navigable via the MANATEE web interface [57], which interacts with the annotation database. Gene products were assigned descriptive names based on database matches to gene products and protein domains that have been functionally characterized to avoid problems commonly associated with circular annotation. Through MANATEE, annotators were easily able to access the computationally derived data in a compact summary page. Many of the sections in the primary MANATEE display page link out to supplementary pages with more detailed information or specific analysis results, such as alignments and external database descriptions.

A set of naming guidelines (see methods) was adopted to provide consistency in functional annotation, but the variable nature of gene families and types of evidence available make it difficult to mandate exact nomenclature. A critical component of the effort was regular communication and discussion of specific annotation examples among the annotators. Choices among multiple possible names and occasional exceptions to the guidelines were made based on consensus decisions by the annotation group as a whole.

#### Protein families

*Arabidopsis *proteins were classified into protein families to facilitate and enhance their functional annotation. The identification of putative protein families enables visualization and navigation of relationships between proteins and allows annotators to curate related genes consistently and accurately as a group. Once all the gene structures had been examined, annotators reexamined family members to ensure that members were consistently and appropriately named within the family context.

Classification of proteins into families should produce clusters of proteins with common evolutionary history and sequence similarity and hence similar biochemical function [58,59]. There is no single standard for the classification of protein families [60-62]. Our approach is based upon conserved domain composition, taking into account both previously identified domain signatures in Pfam [12] and TIGRFAM [13] and any remaining potential novel domains identified in the *Arabidopsis *proteome using independent methods (see Methods). Our protocol differs significantly from the homology-based approach used to calculate paralogs for the *Arabidopsis *complete genome publication [1], which relied on BLASTP matches between proteins with an E value <1e-20 and extending over at least 80% of the protein length. A benefit of our approach is that related families are easily identified by the fact that they share one or more domains.

**Figure 3:**
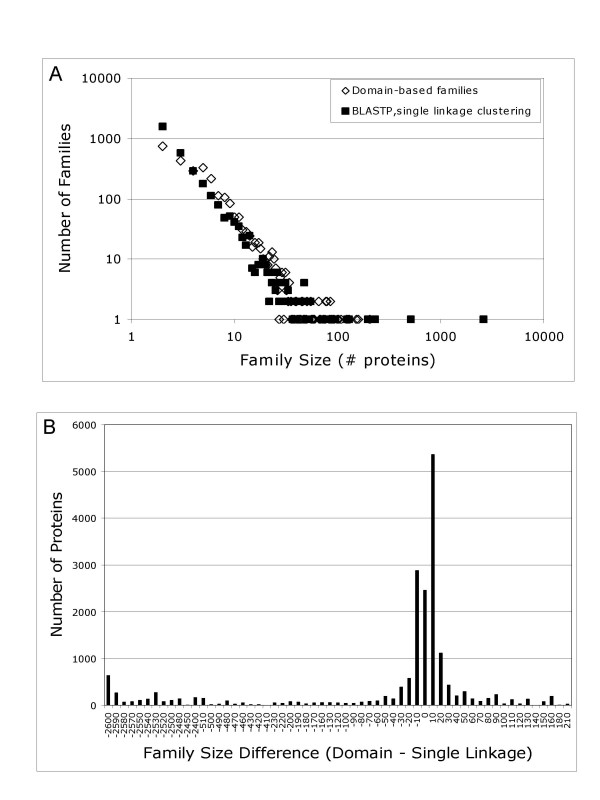
Distribution of proteins within families constructed using two distinct family building methods: our currently employed domain composition based clustering versus the single-linkage BLASTP-based clustering method originally described.A: Frequency distribution of family sizes created by the two methods. B: Difference between the two methods evaluated at the protein levelon a per protein basis. The difference in family size between domain-based clustering and the single-linkage clustering method (DBC – SLC) was calculated for each protein that was included in a family using both methods. The histogram shows the total number of proteins found at each size difference displayed on the abscissa, binned at increments of 10.

Using our domain-based protein classification and family construction methods, 18,641 (71%) of the *Arabidopsis *gene products are classified as members of 2,691 protein families [63]. On average, a family contains 7 members, although large families of kinases, transducins, zinc finger proteins, hydroxyproline-rich glycoproteins, myb family transcription factors, and cytochrome P450s are each represented by more than a hundred proteins and altogether comprise approximately 5% of the proteome. By contrast, the BLASTP method with single linkage clustering produces 18,260 proteins built into 3,142 families. A comparison between the results of protein family building using our domain-based classification scheme and our original BLASTP-based clustering approach is shown in Figure 3. Figure 3A shows that the distribution of protein families according to size produced by the two methods is quite similar overall. Figure 3B illustrates the differences in family sizes built by the two methods on a per protein basis.

While most of the proteins in domain-based families are clustered into families of about the same size using single linkage clustering (SLC), this latter approach can produce anomalously large families. For example, the largest SLC family contains 2601 proteins. Using domain-based clustering (DBC) this same set of proteins resolves into 216 families ranging in size from 205 to 2 members. While the largest fraction of genes in the SLC family are protein kinases, other families such as cytochrome P450s, PPR-repeat proteins and calmodulins are included with each group, being linked by sequence similarity to only a subset of the other groups of proteins in the family. These families are well-resolved by the DBC method. Conversely, the SLC method can also produce fragmented families and singletons. This occurs where the functional domain covers only a small percentage of the overall protein size, as for example with many DNA binding and protein interaction domains. While the DBC method groups together proteins with these relatively small domains, the criteria of sequence identity and match length required by SLC is only fulfilled for small subsets of proteins within the domain-based families. For example, one DBC family of 151 members, which represents proteins with a single zinc finger (C3HC4-type RING finger) family domain (PF00097), is split by SLC among 32 families ranging in size from 14 to 2 members and 25 singletons. Clearly there is great diversity in this group of proteins that form a DBC family on the basis of a relatively short domain. However, this can be a useful grouping when no other information is available.

The DBC method also over-fragments families under different circumstances. A set of paralogous proteins can contain some members that hit PFAM domains above the trusted cutoff, and some that do not because of divergence and/or lack of plant representatives in the PFAM seed. This results in the creation of *Arabidopsis*-specific domains that are, in effect, redundant with PFAM domains but are considered distinct, causing inappropriate fragmentation of families. For example, there are 17 proteins in a single SLC cluster that contain the "seven in absentia" (SINA) domain (PF03145), but two of these score just below the trusted cut-off. This results in the creation of 3 DBC families of 10, 5, and 2 proteins respectively. The Pfam domain profile can be retuned to include the missing *Arabidopsis *representatives and remedy any over-fragmentation resulting from the insensitivity of the original domain profile (data not shown).

Overall, close to 60% of clustered proteins fall into families whose sizes differ by fewer than 10 members between the two methods of family construction. The domain-based approach produces fewer, slightly larger families, and some anomalously large families are eliminated.

#### Duplicated genes (segmental and tandem duplications)

The large scale duplications of the *Arabidopsis *genome have been extensively analyzed and documented ([64-66] and references therein). In addition to analyzing genes in the context of gene families, a further analysis of gene names was performed in the context of duplicated genes that may share similar or identical functions. Using approaches and criteria similar to those employed by others, we developed tools to facilitate the identification of segmental and tandem duplicated genes in our latest annotation (web resources at [67,68]). We identified 6,582 protein-coding genes within the segmentally duplicated regions of the genome and 3,737 genes within tandem duplications some of which are found to be within the segmentally duplicated regions. In all, there are 9,533 presumed paralogous protein-coding genes, representing 36% of the *Arabidopsis *proteome. We then examined the functional annotation of these paralogous groups, verified the uniformity of their annotations and manually resolved any inconsistencies.

#### Gene ontology

In order to maximize the usability of the annotation data set, *Arabidopsis *protein-coding genes were further classified using the controlled vocabularies of the Gene Ontology (GO) [69]. TIGR is a member of the GO Consortium [70], a collaborative international effort to organize and define gene products using standard, species-independent terminology. GO is now widely used in plant, animal and microbial genomics and has become one of the principal tools employed in the annotation of genes and their products [71-74].

GO consists of dynamic, controlled vocabularies describing three areas of biological systems: molecular function, biological process, and cellular component. Each GO annotation is required to contain an evidence code describing the type of evidence that supports it [75]. The evidence types used in manual GO curation range from direct experimental evidence and published inferences based on experimental data, to annotator inferences from examination of sequence and domain similarities.

GO terms were assigned to *Arabidopsis *gene products based on similarity to functionally characterized proteins and/or functional domains. The majority of the *Arabidopsis *GO associations fall into the ISS category (inferred from sequence or structural similarity) since there was no published experimental evidence available. These inferences were made by assessing all of the similarity evidence available, including BLASTP results, HMM search results, Prosite and Interpro membership, protein family relationships, and similarity to other gene products having GO annotations. Proteins that were examined and had either weak or partial similarity to functionally characterized proteins were deemed to have too little evidence to warrant functional GO assignments and were given the GO term "unknown". This term exists so that annotators can capture the fact that they looked at the evidence available for a specific gene product and could make no assertion about the role this gene product might play in the organism.

At TIGR, all GO assignments to *Arabidopsis *genes were performed manually with emphasis on molecular function terms, but assignments to biological process and cellular component terms were added when they could easily be inferred from the evidence considered. This work was carried out in coordination with scientists at TAIR [76]. We regularly integrated the manual GO curation provided by TAIR into our dataset in order to minimize redundancy of effort between institutes. However, TAIR associations made automatically through purely computational methods were excluded from our dataset. Of the 49,505 distinct curated associations between 26,207 *Arabidopsis *genes and GO terms in the final release, 6,424 associations were contributed uniquely by TAIR, 25,131 loci are annotated with at least one TIGR association, and 4,642 loci are annotated with at least one TAIR association, with 3,566 of these annotated by both centers.

Leaving aside the specific GO category "unknown", 29,773 specific GO terms are assigned to 14,529 genes. Of these, 17,259 terms (assigned to 13,070 genes) are molecular function, 8,864 terms (7,111 gene assignments) are biological process, and 3,650 terms (3,257 gene assignments) describe cellular component. The GO function term "unknown" was assigned to all other genes after confirming the lack of other evidence. The decrease in the proportion of genes with a meaningful GO assignment (55%) compared with the number of genes given a functional assignment at the time of genome completion (69%; [1]) is most likely a reflection of the more rigorous and uniform standards applied during our whole genome reannotation effort

**Figure 4:**
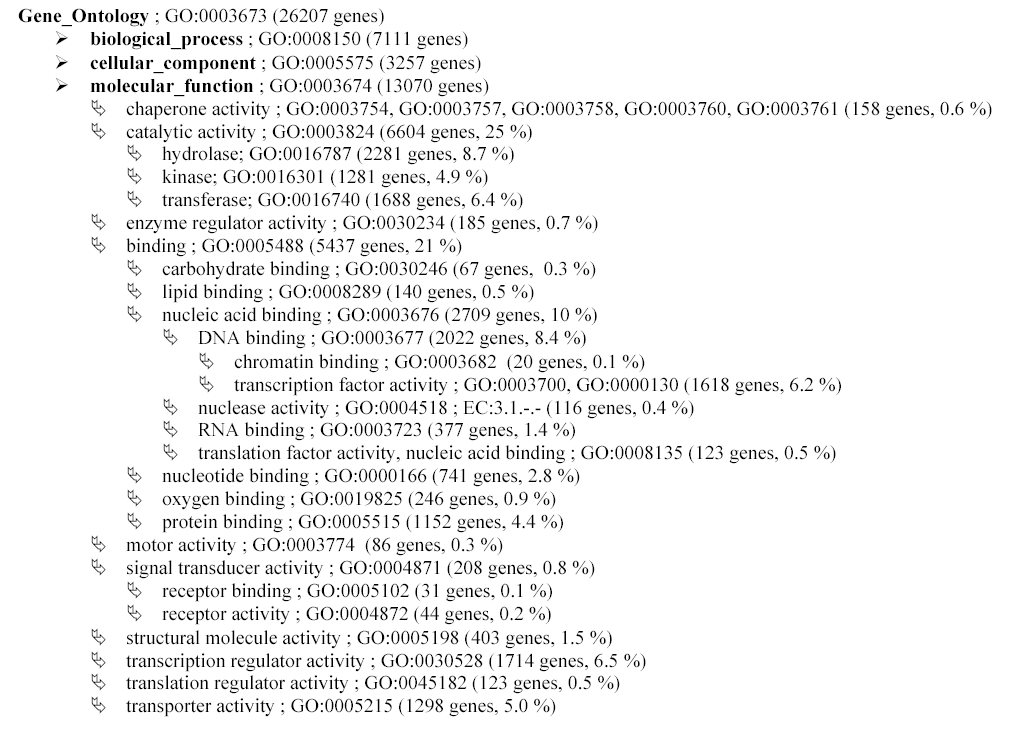
The distribution of genes in major categories of the Gene Ontologies. Each of the 26,207 protein coding genes was assigned to at least one GO term, with our primary focus the assignment of genes to Molecular Function terms. The ontology categories illustrated correspond to those of the plant GO slim obtained from

As a result of the reannotation effort, each protein-coding gene in the genome has been manually assigned to at least one GO term (data available at [77]). Figure 4 provides a summary of the current state of functional characterization of the *Arabidopsis *genome. Among the most abundant functional role categories, 25 % of the genes are assigned catalytic functions including hydrolase, kinase, or transferase activity; 10 % bind nucleic acids, primarily DNA, including the 6.5 % categorized as transcription factors; 4.4 % are categorized as protein binding, many inferred from the presence of domains implicated in protein-protein interactions such as the RING Zn-finger [78] and leucine-rich repeats [79]; and 5 % are classified as transporters.

### Transposable element and pseudogene annotations

Transposons and pseudogenes were the last categories of gene models to be systematically addressed by the re-annotation process. Many gene models with similarity to transposons or transposon-related proteins were originally annotated as protein-coding genes. However, the majority of these regions are degenerate, making it difficult or impossible to model ORFs across their entire extent, although shorter ORFs with similarity to parts of transposons may be contained within the boundaries. Thus, the legacy annotation for transposon-related sequences consisted of a mixture of genes and pseudogenes.

In release 5.0, all transposon-related sequences were uniformly classified by searching the entire genome against a curated database of protein-coding transposon sequences [80] using the dps alignment utility of the AAT package and automatically applying the corresponding transposon family annotation. Each transposon-related region was defined by a single pair of coordinates and classified into one of the major classes of transposable elements as described in [81], shown in Table 4. Release 5.0 contains 2,355 loci annotated as transposons, 1,652 matching retrotransposons and 703 matching DNA transposases and (in contrast to all previous releases) these are no longer included in the count of "protein coding genes" nor are they represented in that dataset. It should be noted that our transposon annotation has been restricted to elements with protein coding potential. Assimilation of the smaller elements and other classes of repeated sequences into the genome annotation remains a task for the future.

Like transposons, pseudogenes are difficult to annotate accurately in an automated manner. Different gene prediction programs will often generate predicted gene structures that are dissimilar to each other and inconsistent with the homologous sequence alignments, introducing introns to circumvent frameshifts and premature stop codons. Pseudogenes are often detected during manual curation of these gene predictions, because the gene model cannot be modeled consistently with homologous protein alignments due to sequence degeneracy that results in stop codons that interrupt the open reading frame. Pseudogenes are often found in transposon-rich regions such as those associated with the pericentromeric regions. In our annotation, pseudogenes, like transposons, are described simply as a single pair of coordinates (5' and 3' ends) that span the genomic region in which they are found, and are classified on the basis of sequence homology to known proteins. In the current release, 1,431 loci are classified as non-transposon-related pseudogenes, of which approximately one third are similar to genes of known function. These include kinases, disease resistance proteins, ribosomal proteins, and others found in large gene families in *Arabidopsis*. The remaining pseudogenes are similar to proteins from *Arabidopsis *or other species that have no known function and likely represent degenerate genes of hypothetical proteins yet to be characterized. Like transposons, the majority of pseudogenes in the current annotation were named by an automated process.

## Conclusion

With respect to the annotation of gene structure and gene function, our reannotation effort has focused mostly on the protein-coding subset of all *Arabidopsis *genes. This reflects a combination of community interest (knowing the entire gene repertoire of an organism) together with databases and gene prediction programs that are relatively effective in identifying and delineating such genes. Without a doubt, the largest contribution to improved gene structure annotation over the last three years has been the generation and release of FL-cDNA sequences by Ceres Inc. [17], by the RIKEN-SSP collaboration [19,41] and by the INRA-Genoscope group [21]. However, because of the bias to annotate genes with presumed functional ORFs, there are likely many genes for regulatory and non-coding RNAs in addition to those already described [82-84] that remain to be discovered and incorporated into the annotation.

Although the accurate annotation of transposable elements is important, our approach was simply to comprehensively identify regions of the genome with homology to transposon ORFs and to explicitly differentiate these from the remaining protein-coding plant genes. More work is needed in this area to improve the resolution and depth of annotation for these complex features, including the deconvolution of polyprotein ORFs, classification of complete, fragmented and degenerate elements, and delineation of repeat structures including long terminal repeats, direct repeats and insertion sites.

With this final release from TIGR, primary responsibility for maintaining and updating the *Arabidopsis *annotation in North America has been assumed by TAIR. It can be anticipated that the annotation will continue to be both improved and enriched. One important distinction between the annotation processes at TIGR and at TAIR is that the former has been entirely sequence-based. This is to some extent historical but also reflects our philosophy that DNA sequence is a public, unambiguous and easily exchanged data type that can for the most part be incorporated into annotation using computational tools. Looking ahead, additional sequence information will permit the refinement of gene structures, while the functional annotation will be enriched both by the availability of new experimental data and by TAIR's policy of including results from expression and other kinds of analyses to characterize each gene and its function fully.

## Methods

### The TIGR genome annotation pipeline, gene modeling and gene processing

Prior to beginning our reannotation effort, we incorporated the remainder of the *Arabidopsis *genome into our relational database (ATH1) as BAC sequences and annotations derived from the sequencing centers, the MIPS database, and GenBank. The annotation associated with these sequences provided the substrate for annotation improvements. Each BAC sequence was run through our eukaryotic annotation pipeline called Eukaryotic Genome Control (EGC). This pipeline consists of a series of steps during which bioinformatics tools are applied to the genomic sequence. The *Arabidopsis *EGC pipeline consists of a single Makefile run nightly on a Linux server. The Makefile runs a series of Perl scripts, each a wrapper around a bioinformatics tool responsible for launching an analysis (e.g. BLAST search), parsing the results, and loading the results into ATH1.

The pipeline manages two primary tasks: processing the bare genome sequence and processing the individual genes and gene products. The genome sequence processing involves several aspects of gene identification and the gathering of evidence for gene structures. Statistical gene finders including GENSCAN+ [54], GeneMark.hmm [55], and GlimmerA [56] are run to gather gene predictions. The GeneSplicer [85] splice site prediction tool is also run to highlight potential splice sites along the genomic sequence.

Transcript and protein spliced alignments provide our greatest resource for accurately identifying and modeling genes, often complemented by the gene predictions described above. We rely heavily on the AAT package [86] to identify genes and resolve gene structures using transcript and protein alignments, and this represents a primary component of EGC. While several other tools exist for generating spliced alignments between transcript sequences (ESTs and FL-cDNAs), including sim4 [87] and BLAT [88], they were not designed for aligning spliced transcripts of diverged species, but rather for accurately mapping near-identical transcript sequences. The AAT package (dds/gap2), although significantly slower than sim4 and BLAT, can generate alignments to divergent transcript sequences. The complete repertoire of TIGR Gene Indices, which includes 22 different plant species, were aligned to each of the *Arabidopsis *BACs at the nucleotide level using the dds-gap module of the AAT package, providing a great wealth of evidence for identifying conserved plant genes and resolving gene structure components (example shown in Figure 2). The AAT package also includes tools (dps/nap) for aligning related protein sequences to the genome, taking into account splice sites and resolving intron/exon boundaries via protein spliced alignments. TIGR's in-house non-redundant protein database (NRAA) was searched and aligned to the *Arabidopsis *BACs using this tool. The AAT package is available at [89].

Following genome sequence processing, the second stage of EGC – individual gene processing – begins. For the comprehensive reannotation of the *Arabidopsis *genome, all the initial gene structure annotations were derived from the first pass annotation of the completed genome [1].

Each gene annotation is subjected to a series of bioinformatic analyses including:

• WU BLASTP [90] search of NRAA.

• Pfam [12] and TIGRfam [13] search using HMMER2 [91]

• Search of PROSITE, PRINTS and ProDom, followed by Interpro classification including the results from the Pfam and TIGRfam searches using InterProScan [92].

• Transmembrane domain identification using TMHMM [14].

• Cellular localization prediction using TargetP [93].

• Signal peptide prediction using SignalP [94,95].

To ensure that the gene-based searches always reflect the most current gene structure, genes that have been structurally altered during our reannotation were targeted each evening by EGC and reprocessed to gather the latest bioinformatics data.

### Computing protein families

To identify domains in *Arabidopsis *peptides, the proteome was searched against Pfam and TIGRfam HMM profiles using HMMER2. Any sequence region scoring above the trusted cutoff assigned to the domain profile was designated as representing that domain. These domain sequences were then removed from the protein sequences and the remaining peptide sequences were searched against each other using BLASTP for subsequent clustering and alignment in order to identify potential novel domains not represented in the domain databases. Similar peptide sequences were clustered by creating a link between any two peptide sequences having an identity above 30% over an amino acid span of at least 50 aa. and an Expect value < 0.001. The Jaccard coefficient of community [96] was calculated for each linked pair of peptide sequences *a *and *b *as follows:



with the Jaccard coefficient (J_a,b_), which we also refer to as the link score, providing a measure of similarity between the two proteins. The associations between peptides that had an insufficient link score were dissolved, and the remaining links were used to generate single linkage clusters. The clustered peptides were then aligned using ClustalW [97] and used to develop conserved protein domains not present in the Pfam and TIGRfam databases. *A. thaliana*-specific domain alignments containing five or more members were considered true domains for the purpose of building families. The peptides in alignments were searched back against the *Arabidopsis *proteome to seek out additional members that may have been excluded during earlier stages due to the parameters employed.

Full length protein sequences were then grouped on the basis of the presence of Pfam/TIGRfam domains and potential novel domains. Proteins with exactly the same domain composition were then classified into putative protein families. The protein family classifications resulting from our analysis are available at [63].

### Gene name curation protocol

The following naming conventions were developed and followed with only rare exceptions:

1. If a gene product had an identical match to a functionally characterized protein, then the gene product was given the name of the characterized protein.

2. If the characterized protein had previously been given a symbol, the symbol was incorporated into the name in parentheses at the end of the name (e.g., holocarboxylate synthetase 1 (HCS1)). Note that the prefix "At," for "A. thaliana," present in some gene symbols in the literature, was omitted since it is redundant. When a functionally characterized protein had multiple names, or aliases, these were included, separated by '/' (e.g., phytochrome A specific signal transduction component (PAT3) / far-red elongated hypocotyl protein 1 (FHX1)). In most of these cases, the first name is typically the functionally characterized name followed by the original gene name. While there was a concerted effort to associate aliases and gene names to a particular locus, inevitably some names may have been missed.

3. If a gene product was not functionally characterized but had a significant match to a functionally characterized protein and was thus believed to be functioning as that protein, then the gene product was designated as putative (e.g., arginine-tRNA ligase, putative). In most cases the Swiss-Prot name was used when there were naming inconsistencies. As in the naming for characterized proteins, aliases were included when they existed.

4. When a gene product had a significant domain hit or partial yet significant characterized protein matches, or belonged to a characterized family but did not have significant homology to family members that had been functionally characterized, the protein was given the domain or family name and designated as a family member (e.g., DNA-binding family protein). In some cases, the significant domain hit did not imply a function for the gene product; these proteins were named for the domain, but designated as domain-containing proteins (i.e.: DC1 domain-containing protein). In cases where there were no significant domain matches and the gene product had either weak similarity or partial similarity to functionally characterized proteins, gene products were named as the protein but given a "-related" designation (i.e.: cysteine protease-related).

5. Many gene products did not have significant matches to characterized proteins or domain hits and functionality could not be deduced. In such cases, a gene product supported by EST and/or cDNA evidence was designated as an expressed protein, while those supported by gene prediction only were designated hypothetical.

### Identification of duplicated genes within chromosome segmental duplications

All-vs-all BLASTP searches were performed for the entire set of protein coding genes. These results were analyzed in the context of chromosome positions, applying a Waterman-Eggert-like alignment algorithm [98] to ordered gene lists. A Java based dot-plot viewer was developed to facilitate the identification and analysis of syntenic or duplicated regions inferred from BLAST matches between pairs of genes, providing rapid visualization and navigation of the data. The viewer includes user-specified filters to exclude matches based on the number of matches or E-value desired (software available at: [99]; note that the viewer has been subsumed by the DAGchainer distribution [100]). Using this tool, the list of tentatively duplicated gene pairs was refined and additional regions were identified manually. The curated list of segmental gene duplicates can be found at [68]. The data are mostly consistent with those reported previously [65].

### Identification of tandemly duplicated genes

Tandemly duplicated genes were identified as described previously [1]. Neighboring genes were analyzed along each chromosome, and gene pairs having an E-value <= 1e-20 and separated by not more than one unmatched gene were classified as tandem duplicates. An array of tandem duplicates was allowed to have only one unrelated member within the array. The list of tandem gene arrays can be found at [67].

### Specification of sequence overlaps between adjacent BACs in the tiling path and chromosome construction

The tiling path for the *Arabidopsis *genome describes the order and orientation of the BACs, YACs, cosmids and other pieces of DNA that collectively represent the sequence of the entire genome. To represent the BAC tiling path, we used a well-known data structure called a double ended queue. Each BAC was represented by a single node in the queue with pointers to the preceding and succeeding BAC. Each node contained additional attributes including the orientation of the BAC sequence, an indication of an overlap or gap between each adjacent BAC, the size of the overlap in base pairs, and the size of any terminal non-overlapping sequence from the overlapping regions to the BAC termini. Each node with pointers was described textually by a single row of a table which exists in ATH1, our *Arabidopsis *annotation database.

Chromosome sequences were constructed by joining the regions of BAC sequences according to their orientation and position of overlap, envisioned as single *in-silico *recombination events between the overlapping regions of BAC pairs. One of the major problems in building (and re-building) the composite sequence from the constituent BACs and other molecules is inconsistency of sequence between the two elements of the overlap. Part of this may be due simply to mutations in the BACs sequenced or to sequencing errors. These inconsistencies can lead to different models for the same gene on the two BACs and make merging of these inconsistencies into a single whole genome annotation very difficult to automate. To minimize the amount of poor quality sequence in the chromosome representations and to better automate future builds, we developed the concept of "high quality overlap regions" (HQORs).

We define an HQOR as a genome sequence region found to align perfectly between two adjacent overlapping BACs. Candidate sequences to represent HQORs were identified using MUMMER [101,102], and a provisional HQOR was chosen as the longest aligned region of perfect sequence identity. To verify the quality of the overlapping region flanking the provisional HQOR, the flanking regions were aligned and assessed using GAP [103]. If use of the provisional HQOR in the chromosome build would result in the incorporation of the model-corrupting base(s) into the sequence, the MUMMER alignments were re-examined and a different HQOR was identified, the use of which would circumvent this problem by shifting the point at which the recombination is made between the overlapping BAC pair. If the provisional HQOR resulted in long flanking sequences within the presumed overlap with low levels of identity suggesting an incorrect automated specification of the overlap, the MUMMER output was reexamined to identify other candidate HQORs that more accurately portray the tiling. This final step addresses potential problems caused by the presence of identical repeats near the ends of the BACs.

After constructing each chromosome sequence from the BAC tilings, the coordinate positions of the BACs within the chromosome were utilized in order to copy all BAC annotations to the chromosome with the appropriate coordinates. The BAC tiling data as described here are included in our XML-based data release [22], and navigable from [104].

## Authors' contributions

BJH was responsible for database content and many aspects of data analysis and drafted the manuscript. JRW carried out data analysis and data integrity checks, and assisted in user interface design and implementation and in the drafting of the manuscript. CMR, LIH, RKS, RM, MF and APC performed manual curation of gene structures and functions, including GO assignments and in manual evaluation of computational pipeline outputs. CY developed code for identifying HQORs and for building the pseudomolecules from BACs and for manually curating the tiling path for the entire genome. DW developed and implemented the protocols for generating *Arabidopsis*-specific protein domains and building paralogous families for facilitating annotation. ORW was involved in database design and the design and implementation of the annotation computational pipeline. CDT contributed to data acquisition, interpretation and analysis and to the drafting of the manuscript. All authors read and approved the final manuscript.

## Figures and Tables

**Table 1:** Statistics for *Arabidopsis *reannotation Release 5.

	Chr. 1	Chr. 2	Chr. 3	Chr.4	Chr. 5	Total
**DNA molecules**						
Length (Mb)	30.269	19.702	23.465	18.582	26.978	118.998
%GC						
overall	35.9	35.9	36.3	36.2	35.9	36.0
coding	44.1	44.2	44.3	44.2	44.1	44.2
intronic	32.4	32.3	32.6	32.4	32.3	32.4
intergenic	30.8	31.4	31.6	31.6	31.1	31.2
						
**Genes**						
# genes	6,772	4,104	5,233	3,985	6,113	26,207
gene density (kb/gene)	4.47	4.80	4.48	4.66	4.41	4.5
Avg. gene length (bp)^a^	2,287	2,156	2,197	2,269	2,227	2,232
Avg. protein length	425	398	417	421	419	417
# genes in protein families	4,834	2,884	3,803	2,839	4,281	18,641
#genes duplicated via segmental chromosome duplications	1,868	961	1,315	1,147	1,291	6,582
#genes found tandemly duplicated	993	545	750	636	813	3,737
#genes with alt splicing isoforms	600	412	444	357	517	2,330
#genes with annotated UTRs	4,717	2,936	3,575	2,724	4,147	18,099
#transposons and pseudogenes	748	817	837	652	732	3,786
# tRNA genes	240	96	93	79	123	631
Exons						
# exons	37,710	21,428	27,937	21,800	33,255	142,130
total length (Mb)	10.378	5.919	7.812	6.011	9.170	39.290
avg exons/gene	5.57	5.22	5.34	5.47	5.44	5.42
avg exon size	275	276	280	276	276	276
						
Introns						
# introns	30,938	17,324	22,704	17,814	27,191	115,921
total length (Mb)	5.060	2.903	3.657	3.016	4.416	19.053
avg size	164	168	161	169	163	164
						
**Proteome**						
# distinct proteins	7,176	4,451	5,540	4,231	6,457	27,855
# proteins with interpro domains	6,142	3,686	4,676	3,573	5,441	23,518
# with TM domain	2,047	1,429	1,599	1,316	1,768	8,159
Signal peptides						
secretory	1,262	797	974	773	1,103	4,909
chloroplast	1,062	681	845	666	1,021	4,275
mitochondria	820	490	612	430	736	3,088

^a^Length of genomic sequence from annotated transcriptional start to stop.

**Table 2:** Summary statistics for TIGR *Arabidopsis *annotation releases.

	Nature (12/00)	Release 1 (8/01)	Release 2 (1/02)	Release 3 (8/02)	Release 4 (4/03)	Release 5 (1/04)
Genome size (Mb)	115.410	116.238	117.227	117.077	119.055	118.998
protein-coding genes	25,498	25,554	26,156	27,117	27,170	26,207
transposons and pseudogenes	NA	1,274	1,305	1,967	2,218	3,786
Genes annotated as alternatively spliced	NA	0	28	162	1,267	2,330
genes with UTRs	NA	4,140	10,219	11,691	17,060	18,099
Protein-coding genes similar to transposon ORFs^a^	NA	487	485	528	531	6
gene density (kb per gene)	4.5	4.55	4.48	4.32	4.38	4.54
exons / gene	5.2	5.23	5.25	5.24	5.31	5.42
average exon length (bp)	250	256	265	266	279	276
average intron length (bp)	168	168	167	166	166	164
Gene structures altered since previous release. (u,a,d,m,s)	NA	-	u: 2,853 a: 690 d: 231 m: 14 s: 167	u:1,366 a: 1,906 d: 221 m: 62 s: 14	u: 2,347 a: 527 d: 143 m: 153 s: 24	u: 2,858 a: 1,393 d: 730 m: 169 s: 28

Gene structure modifications from each previous release are represented by u: updated, a: added, d: deleted, m: merged, and s: split. ^a^Annotated protein-coding genes with a BLASTP match containing an E-value <= 1e-20.

**Table 3:** Genes classified by alternative splicing variation.

**Splice variation classification**	Genes with isoform type	**% alter protein sequence**
Alternative acceptor and/or donor	1,050	70%
Unspliced introns	926	67%
Alternate terminal exons	99	28%
Exon skipping	130	68%
Start or end within intron	520	47%

**Table 4:** Transposon classification.

**Transposable element classification**	**# Annotated genomic regions**
**Class I (Retrotransposons)**	**1652**
gypsy-like retrotransposon family (Athila)	511
gypsy-like retrotransposon family	374
copia-like retrotransposon family	494
non-LTR retrotransposon family (LINE)	264
other	9
**Class II (DNA transposons)**	**703**
hAT-like transposase family (hobo/Ac/Tam3)	77
CACTA-like transposase family (En/Spm)	69
CACTA-like transposase family (Ptta/En/Spm)	127
CACTA-like transposase family (Tnp1/En/Spm)	37
CACTA-like transposase family (Tnp2/En/Spm)	102
Mutator-like transposase family	268
Mariner-like transposase family	9
other	14
